# Practical Notes on Diagnosis and Treatment

**Published:** 1912-08-10

**Authors:** 


### PRACTICAL NOTES ON DIAGNOSIS AND TREATMENT.

**Congenital Pyloric Stenosis.**

Vomiting—obstinate, repeated, and spasmodic, and dating more or less from birth—especially if accompanied by constipation and loss of flesh, should suggest the possibility of congenital pyloric obstruction. The proof of the diagnosis is the recognition of a pyloric tumour and of gastric peristalsis. But—and this is where oversight is frequent—to detect these the child should be examined and watched immediately after the administration of food.

**Fat in Infant Feeding.**

It is sometimes overlooked that in diluting milk, say with barley water, for feeding infants, the proportion of fat in the mixture is defective. Hence an addition of a little cream should be made to each feed; and similarly, to neutralise the deficiency of sugar a piece of loaf sugar should be added.

**Nervous Dyspepsia.**

Rest, change, massage, etc., are the general measures which are recognised as necessary in this condition. As medicinal treatment the following, given in a capsule after food thrice daily, is suitable:—Zinci valerian. grs. iij, acid. carbol. grs. ij, acid. arseniosi gr. $\frac{1}{40}$, ext. cannabis ind. gr. $\frac{1}{4}$.

**Gonorrhœal Bubo.**

This demands rest and the application of the following ointment:—Ointment of mercury, alcoholic extract of belladonna, ichthyol and lanolin. The ointment should be spread in a thick layer on lint, and applied to the bubo; a bandage should then be firmly applied.

**Secondary Syphilis of the Tongue.**

This demands mercury, not iodide of potassium; and it needs mercury locally as well as constitutionally. Mr. Christopher Heath used to recommend perchloride of mercury $\frac{1}{4}$ gr., honey 2 drachms, water to an ounce. A mouthful of this should be held in the mouth for five minutes thrice daily. A mere rapid rinsing out of the mouth is useless.

**Antiseptic Inhalations in Phthisis Pulmonalis.**

The formula advised by Dr. David B. Lees is as follows:—Carbolic acid, creosote, spirit of chloroform, of each ʒij, tincture of iodine and spirit of ether, of each ʒj. A naso-oral respirator, to which a little of the above is added at intervals, should be worn continuously day and night. Recently Dr. Dundas Grant, writing in the *Lancet* on tubercle of the larynx, testifies to the value of the method more especially in its power of checking cough and so promoting local rest for the respiratory apparatus. To overcome the disagreeable odour of the creosote, without interfering with the efficacy of the inhalation, he advises the following:—Creosoti ʒij, ol. pini sylvest., sp. chloroform, aa. ʒiss, menthol grs. x, ol. cinnam. ♏v; or, if cinnamon is objected to, oil of citronella may be substituted.

**Painful Micturition in Young Children.**

This is generally due to excessive acidity of the urine, and can readily be cured by a few doses of potassium citrate. Sometimes the urine will be found to yield a deposit of white urates, and among these may be present some "hedgehog" crystals of sodium urate.

**Epistaxis.**

A ready and effective way of dealing with an attack of epistaxis is to put the patient's feet and legs in a bath of hot water, the temperature of the bath being maintained by adding more water from time to time. To prevent attacks of epistaxis in gouty patients and in patients with high arterial tension, a morning draught of potassium nitrate (10 to 15 grains) with $\frac{1}{2}$ to 1 grain of sodium nitrate should be ordered for daily administration. In the epistaxis of hæmophilia a plug of lint soaked in adrenalin solution has proved successful.

**Arthritis in Ophthalmia Neo-natorum.**

Occasionally joint complications appear in ophthalmia neo-natorum. The prognosis is much better than in the gonorrhœal arthritis of adults. Thorough treatment of the conjunctiva with rest and fixation of the joints is usually followed by prompt improvement.

**Hæmoptysis.**

In some cases in which internal remedies fail, firm strapping of the side of the chest corresponding to the site of the hæmorrhage has proved successful.

**Diabetes Insipidus.**

Some instances of polyuria have been recorded both in acquired and congenital syphilis, and the use of specific treatment has proved successful.

**Hæmorrhage in the Pons Varolii.**

This is distinguished by profound unconsciousness, general muscular relaxation, extremely contracted pupils, Cheyne-Stokes or other irregular forms of respiration, and a rapidly-rising temperature. This last symptom distinguishes pontine hæmorrhage from severe opium poisoning, the other symptoms in the two conditions being very similar. A rising temperature is always of bad omen in a case of cerebral hæmorrhage.

**Progressive Muscular Atrophy.**

The outlook in this disease is often described as hopeless, but there is no doubt that in some cases not only is the wasting stayed but improvement is produced by strychnine. To be of value it is essential that the strychnine be given by hypodermic injection.

**Migraine.**

The use of a seton is rarely resorted to nowadays, but there can be no doubt it is of value in obstinate cases of migraine.

